# Association Between Trauma-Induced Vertebral Fractures and Motor Weakness in Patients With Diffuse Idiopathic Skeletal Hyperostosis

**DOI:** 10.7759/cureus.76403

**Published:** 2024-12-26

**Authors:** Ryohei Saito, Eijiro Onishi, Sadaki Mitsuzawa, Satoshi Ota, Hisataka Takeuchi, Yoshihiro Tsukamoto, Shinnosuke Yamashita, Daiki Sako, Tadashi Yasuda

**Affiliations:** 1 Orthopedics, Kobe City Medical Center General Hospital, Kobe, JPN; 2 Orthopedic Surgery, Kobe City Medical Center General Hospital, Kobe, JPN

**Keywords:** computed tomography, diffuse idiopathic skeletal hyperostosis (dish), magnetic resonance, neurological deficit, paralysis

## Abstract

Background

Diffuse idiopathic skeletal hyperostosis (DISH) is an age-related condition involving abnormal ossification of soft tissues, including ligaments and joint capsules. Patients with DISH have an increased risk of fractures, especially in ankylosed spines, which increases susceptibility to spinal cord injury. This study aimed to explore the risk factors for neurological symptoms in patients with DISH-related fractures.

Material and methods

In this retrospective single-center study, 34 patients with fractures of the DISH-ankylosed segment who underwent surgical treatment were included. Computed tomography (CT) and magnetic resonance imaging (MRI) were used to investigate the fracture type, degree of displacement, rate of spinal canal stenosis, and presence of posterior column injury. Radiographic and clinical risk factors for paralysis, defined as American Spinal Injury Association impairment scale grades A, B, C and D) and sensory disturbances were analyzed. Neurological prognosis was also examined.

Results

Of the 34 patients with DISH-related vertebral fractures treated by surgery, 16 (47%) experienced fracture-related paralysis. The mean patient age was 75.0 years, with 29 men and five women. Four patients (25%) showed postoperative neurological improvements. Injuries up to the posterior column and a high spinal canal stenosis rate on CT sagittal images were risk factors for paralysis. The cut-off values for CT and MRI stenosis rates were 32% and 55%, respectively.

Conclusions

Approximately half of DISH-related spinal fractures result in paralysis. Severe spinal canal stenosis on sagittal CT and MR was identified as a risk factor for paralysis, with cutoff values of 32% and 55%, respectively. In suspected cases of suspected DISH-related fracture, early CT and MRI are recommended. In cases with a high risk of paralysis, early surgical intervention may be indicated to prevent late-onset paralysis.

## Introduction

Diffuse idiopathic skeletal hyperostosis (DISH) is an age-related disease characterized by the ossification of soft tissues, including ligaments and joint capsules. This disease was first described by Forestier and Rotes-Querol in 1950 as senile ankylosing hyperostosis of the spine [1].

In 1975, Resnick and Niwayama et al. defined DISH as a systemic noninflammatory disease characterized by ossification or calcification of at least four consecutive spinal levels [2]. The prevalence of DISH ranges from 2.9% to 42.0% depending on the classification criteria and the presence of risk factors within the studied population [3-5]. Most patients with DISH are asymptomatic; accordingly, DISH is often discovered incidentally on radiography and computed tomography (CT) examination of other diseases [6].

Patients with DISH are characterized by susceptibility to fracture. An ankylosed spine is four times more likely to experience a fracture in its lifetime than a non-ankylosed spine, with a 58% higher risk of associated spinal cord injury [7]. This could be attributed to the high instability of hyperextension (AO Spine-B3) [8] or displacement (AO Spine-C) fractures in patients with DISH [9].

However, the risk factors for neurological symptoms in patients with DISH remain unclear. Accordingly, this study aimed to identify the risk factors for neurological impairment resulting from DISH-related fracture.

## Materials and methods

This retrospective study, which utilized anonymized data with a general opt-out procedure, was approved by the Institutional Review Board of Kobe City Medical Center General Hospital. All procedures performed in this study were in accordance with the ethical standards of our institutional ethics committee as well as the 1964 Helsinki Declaration and subsequent amendments or comparable ethical standards. The requirement for informed consent was waived given the retrospective study design. 

The inclusion criteria of this study were as follows: (1) patients who underwent spinal surgery for vertebral fractures between January 2012 and December 2022 and (2) patients with ossification along the anterolateral surface of at least four consecutive vertebrae, as defined by the Resnick criteria [2]. The exclusion criteria were: (1) patients diagnosed with ankylosing spondylitis and (2) patients who had previously undergone spinal surgery. Based on these criteria, 34 patients were included.

**Figure 1:**
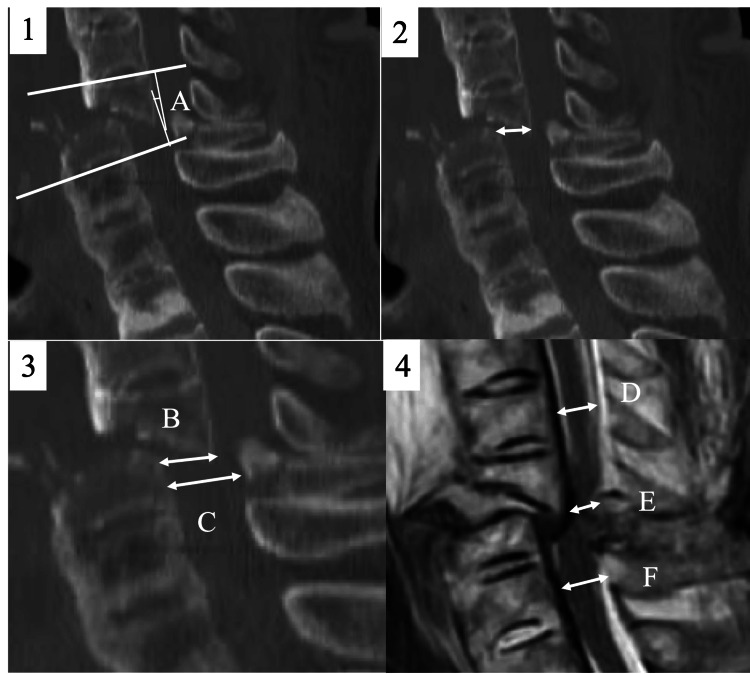
Radiographic parameters 1: Fracture angle: Fracture angle ("A" in figure) was defined as the angle between the superior and inferior margins of the fractured vertebra in the CT sagittal image. 2: Fracture displacement distance (mm): The displacement distance was measured in the slice with the greatest vertebral separation due to fracture in the CT sagittal images. 3: Spinal canal stenosis rate on sagittal CT images: stenosis rate (%) = B/C×100%. 4: Dural stenosis rate on sagittal MR images: dural stenosis rate (%) = ((D+F)/2-E) /(D+F)/2×100%.

We collected data regarding age, sex, body mass index, cause of injury, delay in diagnosis, presence/absence of DISH-related fracture, level of vertebral fracture, posterior-column damage as defined by Denis [10], presence/absence of a complication of ossification of posterior longitudinal ligament (OPLL) at the fracture site, AO classification, and the presence of motor weakness and sensory disturbance as determined using the American Spinal Injury Association impairment scale (AIS) [11]. Paralysis was defined as AIS grades A, B, C, and D. Preoperative magnetic resonance (MR) and computed tomography (CT) images were used to obtain the following data (Figure 1).

The fracture angle was defined as the angle between the superior and inferior margins of the fractured vertebra in the CT sagittal image (Figure 1, Panel 1). The displacement distance was measured in the slice with the greatest fracture-induced separation of the posterior vertebral wall in the CT sagittal images (Figure 1, Panel 2). The spinal canal stenosis rate on sagittal CT images was calculated as follows (Figure 1, Panel 3):

Stenosis rate $(\%) = \frac{B}{C} \times 100$

To obtain the dural stenosis rate on sagittal MR images, the anteroposterior diameter of the dural canal at the level of the vertebral body above and below the fracture, as well as the anteroposterior diameter of the dural canal at the level of the most narrowing of the fracture, were measured. Subsequently, the dural canal stenosis rate was calculated as follows (Figure 1, Panel 4):

Dural stenosis rate $(\%) = \frac{\frac{D + F}{2} - E}{\frac{D + F}{2}} \times 100$

To identify risk factors for paralysis-associated DISH fracture, we analyzed the correlation of radiographic and clinical parameters with the incidence of paralysis. 

All statistical analyses were performed using JMP17 (JMP, Cary, NC). A p-value < 0.05 was considered statistically significant. After confirming the homogeneity of variance using Levine’s test of equal variances, we performed an independent samples t-test to determine if there were differences in continuous variables according to the presence of neurological symptoms. Data are expressed as mean ± standard deviation. Chi-square tests were performed to determine if there were differences in nominal variables according to the presence of neurological symptoms. Additional multivariate analyses were performed for variables with significant between-group differences. The variance inflation factor (VIF) was used to assess multicollinearity. The receiver operating characteristic (ROC) curve and area under the curve (AUC) for ROCs were obtained by plotting sensitivity against the false-positive rate (1−specificity). The Youden index (J) was calculated to determine the optimal cutoff values for the risk factors. 

## Results

We included 34 patients, comprising 29 men and five women, with an average age of 75.0 years. Motor weakness and sensory disturbance were observed in 14 (41%) and 16 (47%) patients, respectively. Furthermore, 14 patients sustained low-energy injuries resulting from falls from a standing position, whereas 20 patients sustained fractures due to high-energy injuries. Two patients had fractures that were not detected during the initial visit, whereas six patients were not seen on the day of injury (Table 1).

**Table 1:** Demographic characteristics and clinical features DISH, diffuse idiopathic skeletal hyperostosis; BMI, body mass index; OPLL, ossification of posterior longitudinal ligament. Paralysis (+), patients with paralysis; Paralysis (-), patients without paralysis.

Baseline characteristics		Paralysis (+)	Paralysis (-)	p-value
Age, years (mean±SD)		74.2 (47-94)±11.5	75.9 (60-89)±9.8	0.65
Sex, male (n, %)		13 (81.2)	16 (88.9)	0.53
BMI (mean + SD)		24.0 ±6.4	23.7 ±3.6	0.88
Cause of injury	Fall from standing/sitting position	8 (50.0)	6 (33.3)	0.32
	Fall from a height	6 (37.5)	8 (44.4)	
	Traffic accident	2 (12.5)	4 (22.2)	
Delay in diagnosis	Patient delay (n, %)	2 (12.5)	4 (22.2)	0.80
	Doctors delay (n, %)	0 (0.0)	2 (11.1)	0.17
	Interval from injury to hospital visit (days)	0.2 ±0.5	0.8 ±2.2	0.68
	Interval from injury to surgery (days +SD)	22.5 ±44.8	7.8 ±7.3	0.21
Fracture characteristics	OPLL in fracture level (n, %)	4 (25.0)	2 (11.1)	0.29
	Injury of the posterior element (n, %)	11 (68.8)	5 (27.8)	0.02*
Fracture level	Cervical (n, %)	9 (56.3)	7 (38.9)	0.49
	Thoracic (n, %)	6 (37.5)	8 (44.4)	
	Lumbar (n, %)	1 (6.3)	3 (16.7)	
AO classification	A (n, %)	0 (0.0)	2 (11.1)	0.35
	B2 (n, %)	1 (6.3)	2 (11.1)	
	B3(n, %)	8 (50.0)	10 (55.6)	
	C (n, %)	7 (43.8)	4 (22.2)	
Location of fracture	Intervertebral disc(n, %)	9 (56.3)	6 (33.3)	0.09
	Vertebral body(n, %)	7 (43.8)	12 (66.7)	

**Figure 2:**
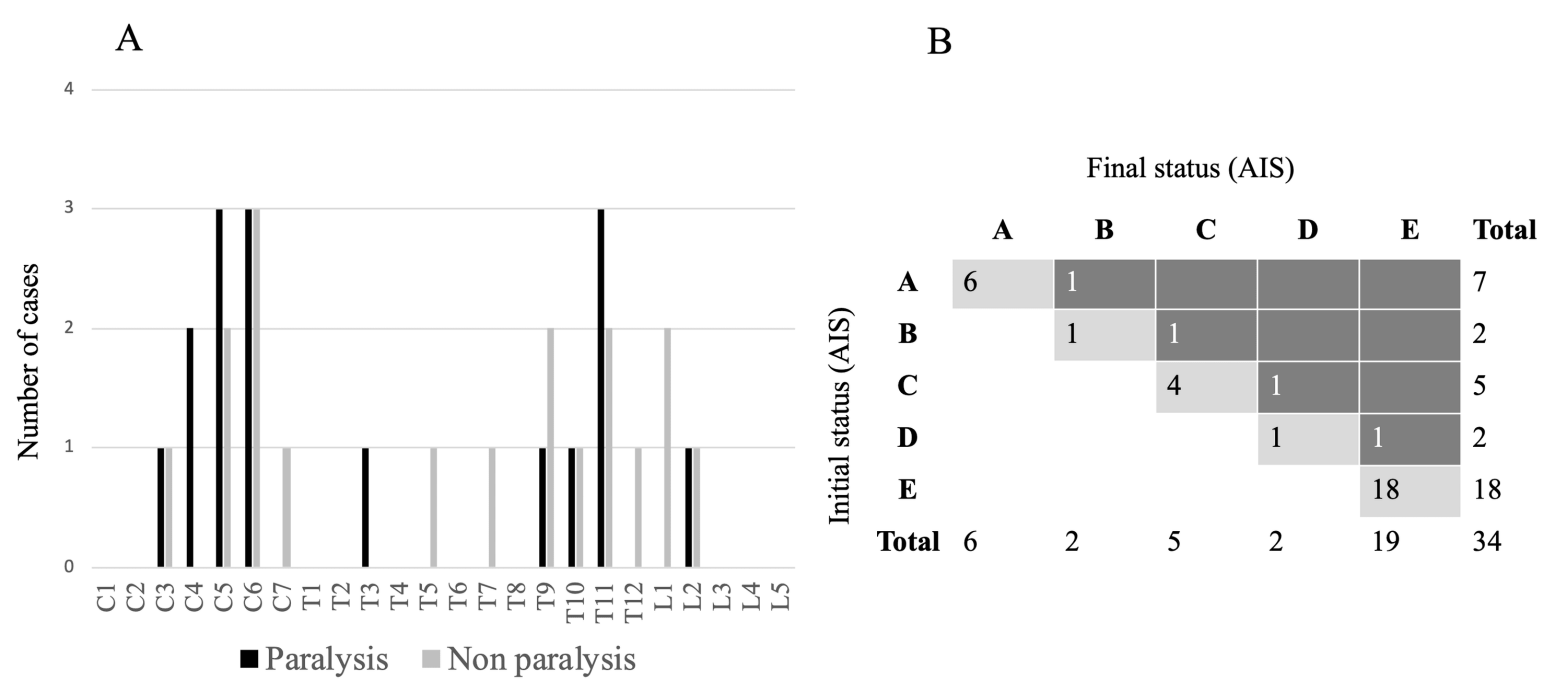
(A) Distribution of fracture level, (B) Neurological prognosis of all patients Panel A: C, cervical; T, thoracic; L, lumbar Panel B: AIS: American Spinal Injury Association Impairment Scale

In addition, 16, 14, and four cases involved the cervical, thoracic, and lumbar spine, respectively. Moreover, 33 patients had fractures that occurred within the ankylosed vertebrae at the DISH, with only one fracture occurring at the lower end of the DISH. Six cases were complicated by OPLL. Injuries up to the posterior column were correlated with paralysis occurrence. The AO classification was A2 in two cases, B2 in three cases, B3 in 18 cases, and C in 11 cases. The group with paralysis tended to have slightly more cervical involvement; however, this difference was not statistically significant. The distribution of fracture levels was bimodal, with a predominance of the lower cervical vertebrae and the thoracolumbar transition area (Figure 2A).

Table 2 shows the correlation between radiographic parameters and paralysis occurrence. The dislocation distance of the fracture and the spinal canal stenosis rate on CT sagittal images and the dura stenosis rate on MR sagittal images were significantly correlated with neurological symptoms.

**Table 2:** Comparison of radiographic parameters according to paralysis CT, computed tomography; MR, magnetic resonance Paralysis (+), patients with paralysis; Paralysis (-), patients without paralysis. *A p-value < 0.05 was considered statistically significant.

Imaging characteristics		Paralysis (+)	Paralysis (-)	p-value
CT sagittal image	Fracture angle (° ±SD)	7.8 ±13.4	6.1 ±9.0	0.67
	Fracture displacement distance (mm ±SD)	9.1 ±4.9	5.4 ±3.3	0.02*
	Stenosis rate (% ±SD)	41.4 ±24.0	14.3 ±17.0	<0.01*
MR sagittal image	Dural stenosis rate (% ±SD)	60.6 ±16.5	39.8 ±15.9	<0.01*

Preoperative paralysis improved in four of the 16 patients, with the improvement being limited to one level based on the AIS grade (Figure 2B).

The multivariate analysis was performed using parameters that showed a significant correlation with the occurrence of neurological symptoms. We observed significant differences in the spinal canal stenosis rate on CT sagittal images and the dura stenosis rate on MRI sagittal images (Table 3).

**Table 3:** Multivariable logistic regression analysis of risk factors for paralysis CI, confidence interval; VIF, variance inflation factor; CT, computed tomography; MR, magnetic resonance *A p-value < 0.05 was considered statistically significant.

Radiographic parameters	p-value	Odds ratio	95% CI	VIF
Injury of the posterior element	0.22	3.65	0.45 - 29.34	-
Displacement distance (CT)(mm)	0.60	1.07	0.83 - 1.38	1.58
Stenosis rate (CT)(%)	0.03*	1.07	1.00 - 1.14	1.58
Dural stenosis rate (MR)(%)	0.04*	1.1	1.00 - 1.21	1.44

**Figure 3:**
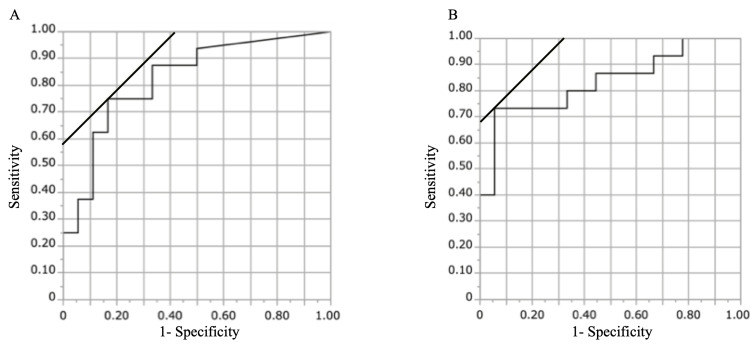
The receiver operating characteristic (ROC) curve A: ROC curve of the spinal canal stenosis rate in the sagittal CT images for predicting paralysis. B: ROC curve of the dural stenosis rate in the sagittal MR images for predicting paralysis.

Univariate logistic regression analysis showed that the area under the curve was 0.82 for CT stenosis rate and 0.83 for MRI stenosis rate, both of which were statistically significant with p < 0.001. The cut-off values for CT and MRI stenosis rates were 32% and 55%, respectively (Figure 3).

These findings suggest that these factors are useful predictors of paralysis due to DISH-related fractures.

## Discussion

This current study investigated the etiology and risk factors associated with paralysis in patients with DISH fractures. The primary findings indicate that approximately half of the DISH-related spinal fractures were accompanied by neurological symptoms, and 41% of the patients exhibited paralysis. Moreover, the prognosis was unfavorable, with only one-fourth of the patients with paralysis showing improvement after surgery. Severe spinal canal stenosis on sagittal CT and MR was identified as a risk factor for paralysis, with cutoff values of 32% and 55%, respectively.

There has been an increase in the diagnostic rate of DISH with the widespread use of CT scans, with previous reports indicating an incidence rate of 7-40% [3-5]. DISH is commonly observed in older men. Long vertebral fusion via DISH results in loss of spinal mobility, and such patients often have low bone density, which makes them susceptible to fracture from even minor trauma [12]. According to a Japanese nationwide multi-institutional survey, the major cause of injury was falling from a standing or sitting position [13]. Once a fracture occurs, the lever-arm mechanism is activated, which increases the likelihood of displacement of the fractured segments and subsequent spinal cord injury. In addition to the physical consequences of spinal cord injuries, there is an increased risk of developing psychological disorders, such as anxiety and depression. These conditions require psychiatric management and follow-up [14]. 

Risk factors for developing neurological symptoms remain unclear. Approximately half of the patients present with neurological deficits immediately after injury, and approximately one-third of the patients present with Frankel grade A-C paralysis [13]. Okada et al. reported that posterior column injuries are associated with structural instability and neurological symptoms [15]. Neurological symptoms are more likely to occur in cervical spine injuries than in thoracolumbar spine injuries, and OPLL complications are a risk factor [16]. Consistent with previous findings, our study shows that injuries extending to the posterior column are associated with the occurrence of neurological symptoms and that cervical spine injuries tend to exacerbate neurological symptoms. This study is among the few that have throughly investigated the association between spinal fractures and post-injury neurological impairment in patients with DISH. In this study, a high spinal canal stenosis rate on sagittal CT and MR images was significantly correlated with post-injury paralysis. Moreover, the cutoff values of the stenosis rate for paralysis were 32% on CT and 55% on MR. CT serves as the first emergency investigation performed when a DISH-related fracture is suspected, which can be used to estimate the risk of paralysis. Delayed palsy has been mainly reported in patients with thoracolumbar fractures with DISH [17]. In high-risk patients, early surgery can prevent delayed paralysis.

In this study, severe spinal canal stenosis on sagittal CT and MR was identified as a risk factor for paralysis, with cutoff values of 32% and 55%, respectively. These values differ for the same sagittal slice. This discrepancy may be attributed to the fact that, in contrast to CT, MR not only assesses bone but also evaluates various soft tissues, including ligamentous components, intervertebral discs, and hematoma, which may increase the degree of compression of the dural canal. In clinical practice, CT is often used for initial screening; however, a comprehensive assessment of paralysis risk can be achieved by combining both imaging procedures of examinations.

Delayed diagnosis is one of the most problematic features of spinal cord injury in DISH-related fractures [18,19]. A previous study showed that patients with DISH may experience various clinical symptoms, including back pain and stiffness in the spinal column [20]. In our study, six patients were not seen on the day of injury because they did not present with serious symptoms immediately after the injury. However, two of these patients developed paralysis symptoms while waiting and ended up visiting the hospital. It is possible that patients with a history of back pain may misinterpret fracture pain immediately following an injury as bruising or chronic pain. In our study, two patients were seen on the same day of injury and were discharged without a radiographic diagnosis on the day of injury, with fractures observed on CT or MRI at the next visit. Furukawa et al. reported that five out of 52 DISH-related fractures could only be noted on MRI, even with retrospective observation [12]. Some reports have recommended CT or MRI to rule out fractures in patients with low back pain who have DISH [17,21-23]. Okada et al. reported that delayed diagnosis contributes to neurological deterioration [13]. Accordingly, prompt CT or MRI is necessary in patients with DISH who present with low back pain.

In addition to early diagnosis, early treatment is necessary in patients with DISH-related fractures. Among the 16 patients who had neurological symptoms, only four showed improved symptoms; further, they were all limited to only one level of improvement in the Frankel classification. Bransford et al. reported that only 22% of patients with cervical DISH-related fractures showed postoperative neurological improvement [24], which does not significantly differ from our findings. Neurological symptoms related to DISH-related fractures are associated with poor treatment outcomes. However, surgical treatment has been associated with a high rate of improvement in neurological symptoms [12]; moreover, early surgery after fracture is associated with neurological improvement [16]. Prompt surgical spinal cord decompression may improve the effectiveness of spinal cord decompression for spinal cord injury.

DISH-related fractures are considered susceptible to displacement given their inherent instability, which is a characteristic similarly observed in long bone fractures [25]. This is also evident in patients with minimal initial displacement without obvious paralysis. Therefore, prompt surgical intervention is recommended for patients with DISH-related fractures, regardless of the presence or absence of paralysis. This is because the risk of paralysis increases when the dislocation exceeds 32% on CT, as observed in our study.

This study has several limitations. First, this was a small-scale, single-center retrospective study. Therefore, the possibility of selection bias could not be excluded, and treatment effects might have been overestimated. Second, this study exclusively focused on surgical cases and did not consider conservative treatment cases. This may have resulted in the inclusion of a considerable number of cases involving paralysis, which may have introduced a degree of bias. Despite these limitations, this study identified novel imaging factors associated with neurological deficits in patients with DISH. In the future, large-scale studies using advanced imaging procedures such as CT and MR will be necessary to provide more accurate predictions regarding neurological symptoms. Furthermore, the integration of artificial intelligence is expected to facilitate the automation of diagnosis, treatment planning, and even prognosis prediction for each patient based on the analysis of patient and image data at the time of injury [26].

## Conclusions

Approximately half of the spinal fractures associated with DISH exhibited neurologic symptoms, with 41% of these cases presenting with paralysis. The prognosis for paralysis resulting from DISH fractures was poor, with only a quarter of patients demonstrating symptomatic improvement following surgical intervention. Severe spinal canal stenosis on sagittal CT and MR images was identified as a significant risk factor for paralysis development, with cutoff values of 32% and 55%, respectively. Patients exhibiting CT spinal canal stenosis greater than 50% were at an elevated risk of paralysis. In cases where paralysis is a high risk, early surgical intervention may be a crucial step in the clinical management plan, provided that an accurate diagnosis is made at the time of the initial examination.
